# Community science datasets identify the spatial occurrence and hotspots of flapper skate ( *Dipturus intermedius* )

**DOI:** 10.1111/jfb.70248

**Published:** 2025-10-09

**Authors:** Danielle L. Orrell, Ciara Wögerbauer, Shane O'Reilly, Thomas K. Doyle, William Roche

**Affiliations:** ^1^ School of Biological, Earth and Environmental Sciences University College Cork Cork Ireland; ^2^ MaREI Centre, Sustainability Institute University College Cork Cork Ireland; ^3^ Inland Fisheries Ireland, Citywest Business Campus Ireland

**Keywords:** capture‐recapture, catch and release angling, common skate complex, conventional tags, time series

## Abstract

The flapper skate, *Dipturus intermedius* (Parnell, 1837), is a large‐bodied, slow‐growing and late‐maturing, Critically Endangered elasmobranch with a constrained population distribution. Here, we use two longitudinal community science datasets to investigate the occurrence of flapper skates in Irish waters. The two datasets are as follows: the Inland Fisheries Ireland mark‐recapture tagging programme (1972–2021; *n* records = 1188) and Irish Specimen Fish Committee records (1958–2022, *n* records = 489). Of the 1677 records, 767 were identified as mature based on size‐based thresholds (*n* males = 418, *n* females = 349). Hotspots of immature and mature individuals were identified across the south, southwest, north and northwest coasts of Ireland. Flapper skates were predominantly caught within 12 NM of the coast in the summer to early autumn (June–October). Time at liberty ranged from 0 to 11.5 years [mean years ± standard deviation (SD) = 1.5 ± 1.6]. Recapture events were typically <50 km from their initial capture location (93% of individuals), with dispersal distances of up to 171 km recorded. This study demonstrates that mature female and male flapper skate, and immature female flapper skate exhibit interannual site fidelity with short dispersal distances and move within existing ICES stock management areas.

## 1 INTRODUCTION

The flapper skate (*Dipturus intermedius*) is a slow‐growing, large‐bodied, long‐lived, low‐fecundity elasmobranch, characteristics that make it particularly vulnerable to extinction (Dulvy et al., 2014; Ellis et al., 2021). Until 2010, this species was grouped under the name of the ‘common skate complex’ (*Dipturus batis*), which has been subsequently identified as the flapper skate (*D. intermedius*) (Parnell, 1837) and blue skate (*D. batis*) (Linnaeus, 1758) (Iglésias et al., 2010; Last et al., 2016). Owing to their morphological similarity and previous grouping under the common skate complex, reliable species‐specific data are limited. Once considered abundant, their population declined in the nineteenth and twentieth centuries and has been attributed to unsustainable exploitation by commercial fisheries (Brander, 1981; Ellis, McCully‐Philips, et al., 2024). Flapper skate is the larger of the two species, reaching ca. 2.5 m in total length (TL) (Last et al., 2016), whereas blue skate has a maximum length of ca. 150 cm (ICES, 2020). With an estimated population reduction of >80% over the past three generation lengths (104 years) (Ellis, McCully‐Philips, et al., 2024), flapper skates are now classified as Critically Endangered under the Irish (Clarke et al., 2016), European (European Commission, 2015) and the global IUCN Red List (Ellis, McCully‐Philips, et al., 2024), as well as listed under the OSPAR since 2003 (under the ‘common skate complex’; OSPAR Commission, 2010). The precarious population status of this benthic species led to its listing as a prohibited species in EU waters since 2009 [Council Regulation (EC) 43/2009, 2025], with mandatory reporting of discards in commercial catches since 2015 [Regulation (EU) 2015/812, 2015]. The flapper skate has a constrained distribution around the UK and Ireland (Ellis, Gordon, et al., 2024), a southerly limit of the Canary Islands (Tuya et al., 2021; described as *D. batis* but later confirmed as *D. intermedius*), a northerly extent of the Norwegian Sea (63° N), and a rare or temporary occurrence in the Western Baltic Sea (Bache‐Jeffreys et al., 2021; Garbett et al., 2023; Lynghammar et al., 2014). Verified egg‐laying sites for the flapper skate are situated off the north and west coasts of Scotland and include Orkney (Phillips et al., 2021) and the Red Rocks and Longay MPA (Scottish Government, 2022). Outside of these areas, reproductive sites remain unverified. Devising effective fisheries management and conservation strategies requires fundamental ecological information, including, but not limited to, spatial distribution, dispersal potential and stock mixing (Bird et al., 2019). Acquiring this information in a marine setting is challenging. However, community science offers a cost‐effective platform that can help generate species datasets over large spatial scales (Earp & Liconti, 2020).

Mark‐recapture tagging is a powerful and low‐cost tool for elucidating the movements of aquatic animals over large spatial and longitudinal temporal scales (Cameron et al., 2019; Cameron et al., 2025; Neat et al., 2015; Simpson et al., 2020). This method involves inserting or attaching a conventional tag either through or just beneath the animal's skin, and each tag has a unique identifier to enable later identification upon animal recapture. Mark‐recapture data have provided insight into the distribution and demography of several skate species in Europe and have been key to ensuring that biological stock units match the spatial scale of management areas (Bird et al., 2019). The Inland Fisheries Ireland's (IFI) (formerly the Central Fisheries Board) Marine Sportfish Tagging Programme was initiated in 1970 to promote catch‐and‐release angling and to gather additional information on the fundamental ecology of elasmobranchs in Irish waters. The benefit of this work was twofold: by encouraging nationwide catch‐and‐release angling, this programme also encouraged volunteer anglers to take a conservation‐minded approach and reduce lethal angling practices. Over the years, the common skate complex has featured on this list as a tagging species.

Founded in 1955, the Irish Specimen Fish Committee is an independent voluntary body that has been verifying, recording, and publicising the capture of large specimen (or ‘trophy’) fish caught on rod‐and‐line by anglers across Ireland. These records encompass both freshwater and marine species, with 27,000 claims spanning 83 unique species submitted since the initiative's conception (Casserly & Roche, 2023). Initially, ‘common skate’ specimens were added in 1958 and reported under a weight‐based threshold that fluctuated between years from 1958 to 1975. In 1976, the common skate was removed from listings owing to a perceived population decline, and it was only recently relisted in 2016 under a total length (TL) based threshold of ≥180 cm.

By examining these two longitudinal community science datasets, we aim to (i) investigate the occurrence and potential hotspots of this Critically Endangered species in Irish waters, (ii) identify potential hotspots of sexually mature flapper skate and (iii) investigate species dispersal using mark‐recapture data.

## 2 MATERIALS AND METHODS

### 2.1 Inland Fisheries Ireland mark‐recapture programme logbook records and tag types

**FIGURE 1:**
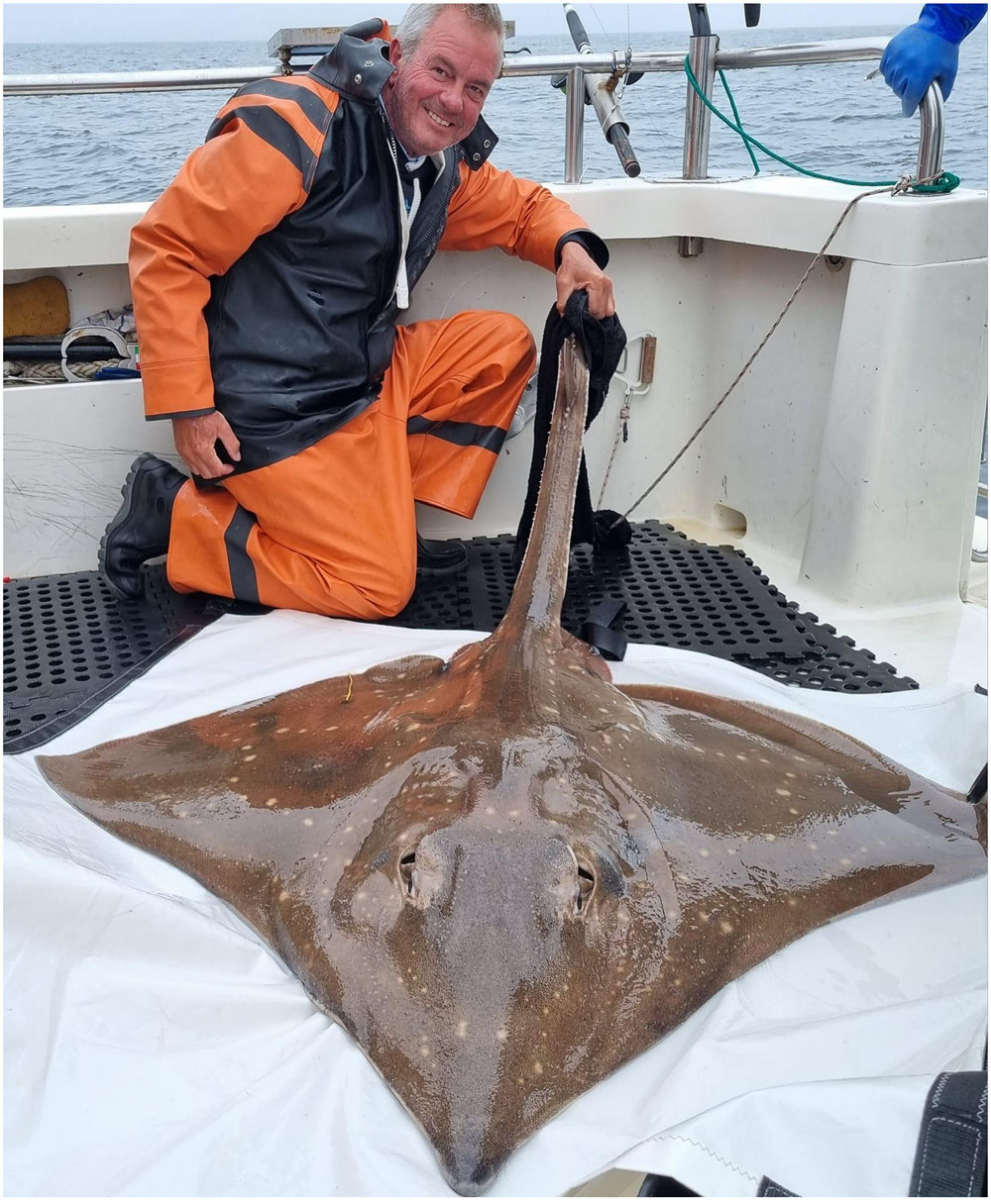
An angler poses with a flapper skate tagged and released as part of the Inland Fisheries Ireland mark‐recapture programme. A yellow dart tag can be seen on the posterior edge of the skate's wing. Photo permission and credit: David Edwards of West Cork Charters.

IFI's Marine Sportfish Tagging Programme (hereafter called the IFI mark‐recapture programme) issues paper logbooks to be populated by volunteer taggers (i.e., angling charter skippers and experienced anglers). Each volunteer is asked to record their name (later assigned a unique numeric identifier), date and time of the tagging event, unique tag identifier, type of tag (jumbo, Peterson, roto, dart), the species, animal sex, TL (measured in cm from the tip of the nose to the end of the tail), wingspan (cm, measured from wingtip to wingtip), estimated weight, a descriptive location of capture, co‐ordinates of capture and any other comments (e.g., whether individuals were multi‐tagged). Over the years, programme participants have tagged skates with one to three mechanical tags in or through the wing and/or tail. Tag types include jumbo tags (1972–2021), Peterson discs (1971–2004), roto tags (2010–2015) and, more recently, dart tags (2018–2021) (Figure 1).

Once paper records are returned to IFI, they are digitised by IFI staff. In cases where a tagger only provides a descriptive location, an estimated position is assigned from a standard co‐ordinate list, which in some cases results in the clustering of capture/recapture locations. Anglers may also choose not to provide specific capture locations and instead provide ports as a proxy to retain personal fishing marks. In some cases, anglers provided fish measurements in inches, which were converted to centimetres during digitisation. The dataset used in the present study included 1188 capture events where the initial capture or recapture fish TL was recorded as ≥160 cm (the size‐based threshold for flapper skates, as seen in Garbett et al., 2023) and a single individual (147 cm) that was later recaptured and exceeded 160 cm. Filtered records spanned 1972 to 2021.

### 2.2 Irish Specimen Fish Committee dataset

Similarly, specimen claims can be made by anglers to the Irish Specimen Fish Committee (hereafter called the ISFC) using a paper form. Form fields include specimen weight, TL, wingspan and sex. Since 2016, a length‐based claim must include a photo of the animal placed on a flat surface (belly down) on a graduated measuring device with an end plate. Additional information includes claimant and skipper name (later assigned a unique numeric code and withheld from public data requests), port of origin, date of capture, capture platform (shore or boat), gear specifications (type of rod and reel, breaking strain of line), tackle type (bait or lure) and a signature from a witness to assure fair angling of the specimen (only mouth‐hooked fish are claimable) and verification of fish weight or length details. Once this record is received, claims are reviewed by the ISFC to ascertain eligibility. Thereafter, successful claims are digitised. In cases where the specific co‐ordinates are not given, an estimated position is assigned from a standard co‐ordinate list based on the port name provided. This standard co‐ordinate list was created independently of the IFI mark‐recapture programme and, therefore, does not match in all cases across the two programmes. Missing sex data were assigned based on the provided photographs (required since 2016).

Only records with the TL provided were used in the analysis, and the dataset was filtered to only individuals with a TL of ≥160 cm, as previously described. A conservative approach was taken to identify duplicate records where volunteer taggers applied a mark‐recapture tag and submitted a specimen fish claim. Any claims where the fish length, date of capture and capture co‐ordinates matched were individually evaluated, and duplicates were removed. After filtering, 489 records remained dated between 1958 and 2022.

### 2.3 Spatial occurrence of flapper skate

Data were manipulated in the R statistical computing package (version 4.4.2; R Core Team, 2024). Records were subsequently visualised in QGIS (version 3.34.15; QGIS.org, 2025) to identify hotspots of flapper skates. To minimise the influence of co‐ordinate accuracy on identifying hotspots, data were aggregated to 50 km^2^ hexagonal grid squares for analysis. Records per grid square were counted using the Count Points in Polygon tool in QGIS. Hotspots are defined as 50 km^2^ hexagonal grid cells with >10% of capture events for a given time frame: all records (1972–2021), records older than 15 years (1972–2006) and records in the last 15 years (2007–2022). The Irish coastline is defined herein by natural features; with the east coast spanning Carlingford Lough to Carnsore Point, the south from Carnsore Point to Mizen Head, the southwest from Mizen Head to Loop Head, the west from Loop Head to Erris Head, the northwest spanning Erris Head to Malin Head and the north from Malin Head to Carlingford Lough.

Records without a classified sex were not used in subsequent sex‐based analyses. In cases where sex was reported on capture but not animal recapture, it was assumed that sex was correctly described. In cases where the classification of sex on capture and recapture disagreed, the sex was assigned as unknown and not used in further sex‐specific analyses. Flapper skates were classified as sexually mature based on the estimates by Thorburn et al. (2023), with males assumed mature at a TL of ≥165 cm and females at ≥203 cm TL.

### 2.4 Recapture events and dispersal distances

Using the IFI mark‐recapture programme data, days at liberty (DAL) were calculated as the number of days between each successive capture event of an individual, with a cumulative DAL calculated for recaptured individuals. For capture incidences where the capture incidences of an individual did not circumnavigate land, the dispersal distance (km) was calculated as a straight line distance in QGIS using the field calculator. For capture incidences where the trajectory of an animal crossed land (*n* = 7), marine distances were calculated using the Fishtracker toolbox workflow in ArcGIS Pro 3.5.1 (Barry et al., 2026). In brief, marine distances were implemented using a combination of ArcGIS Pro (version 2.9.1; Esri, 2021), the ArcGIS Network Analyst extension, Python 3.8 (Python Software Foundation, 2019) and Microsoft Excel. A triangular grid was generated to model fish tracks. The grid was created using the Generate Tessellation tool with parameters optimised for the study area (triangle size of 0.3 km^2^). The polygon grid was converted to lines using the Feature to Line tool, as linear features are required for network analysis. The grid was clipped using the Erase tool to exclude land areas, retaining only the water‐bound features. The Calculate Geometry tool in ArcGIS was then used to determine the distance travelled.

### 2.5 Ethics Statement

Under Irish law, the use of conventional tags (as used by the IFI mark‐recapture programme) to facilitate the identification of individual free‐ranging animals is exempt from the scope of animal welfare legislation. Nonetheless, to ensure animal welfare, the IFI mark‐recapture programme has undergone frequent internal ethical evaluation by the relevant research ethics and compliance officers within IFI, with several refinements to both the tagging methods and specific tags used adopted over the course of the programme. Additionally, participating volunteers are provided with guidance in the form of written materials and video guides on the best practices for handling and tagging of elasmobranchs as part of both the IFI and ISFC programmes.

## 3 RESULTS

### 3.1 Spatial occurrence of flapper skate records

**FIGURE 2:**
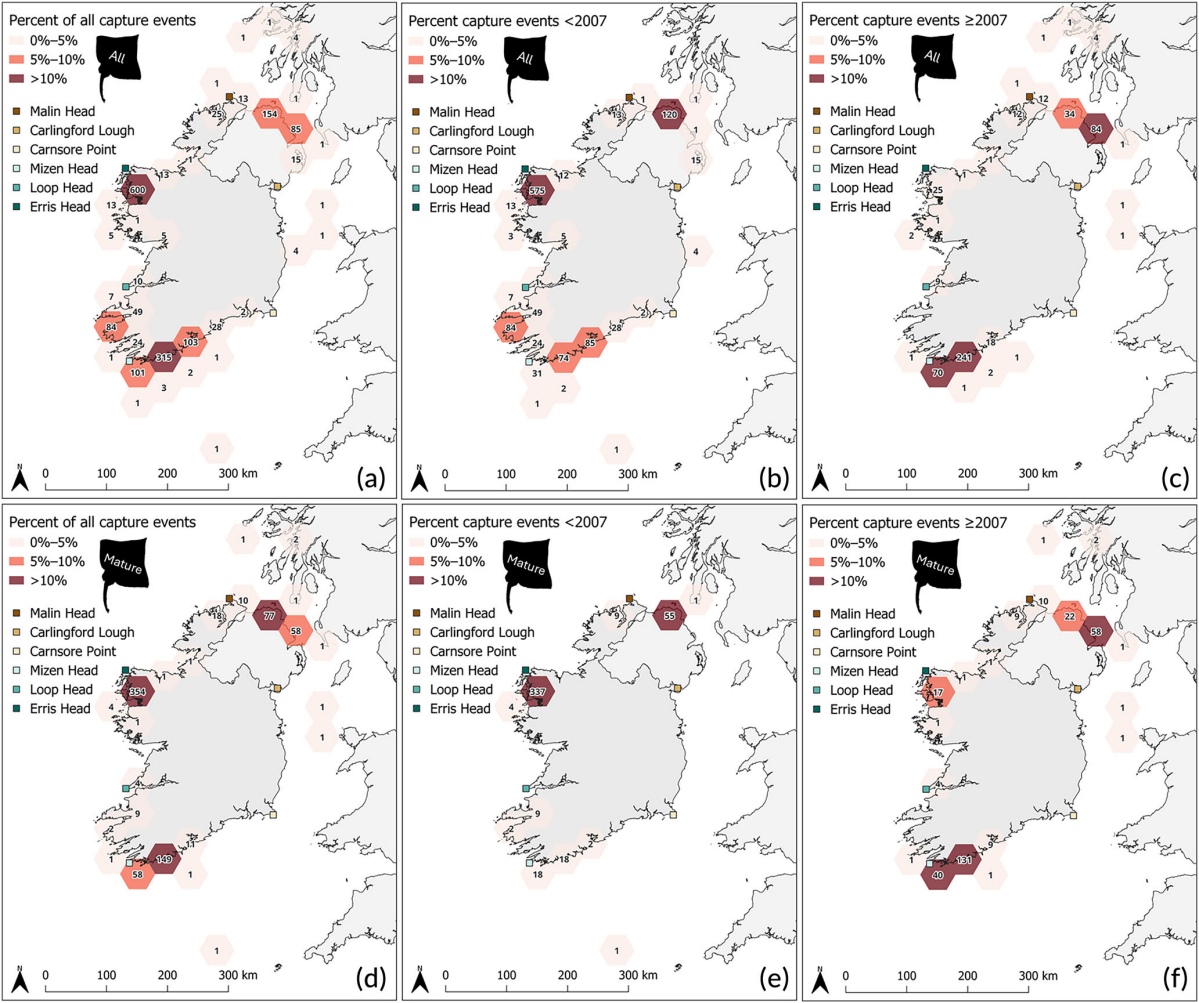
Spatial occurrence of flapper skate (*Dipturus intermedius*) recorded by the Inland Fisheries Ireland Marine Sportfish Tagging Programme and Irish Specimen Fish Committee specimen programme. A 50‐km^2^ hexagonal grid cell is coloured by the percentage of capture records (0%–5%, 5%–10%, >10%) relative to the number of records within a time frame. Cells are labelled as a count of capture events in a corresponding grid cell. (a) All capture records from 1958 to 2022 (*n* = 1677). (b) All capture records from 1958 to 2006 (*n* = 1152). (c) All capture records from 2007 to 2022 (*n* = 525). (d) Mature skate records from 1958 to 2006 (*n* = 767). (e) Mature skate records from 1958 to 2006 (*n* = 456). (f) Mature skate records from 2007 to 2022 (*n* = 311).

In total, 1677 flapper skates (*n* females = 655, *n* males = 432, *n* unclassified = 590) were captured across the IFI mark‐recapture programme and the ISFC specimen fish programme between 1958 and 2022. Most captures were ≤12 NM off the coast by anglers, with only 12 (≤1%) capture records outside of territorial seas (defined as ≥12 NM from the coast). Flapper skate records were reported across all coasts (Table 1); however, the number of contributing taggers was higher on the south coast (*n* = 184) than on any other coastline (*n* taggers range = 36–99). Flapper skate hotspots include the northwest coast in Clew Bay, Co. Mayo (36% of all capture events), and on the south coast in proximity to west Cork (Co. Cork; 19%). Additionally, areas of interest containing 5%–10% of all capture events include two areas along the south coast (6% and 6% respectively), on the west coast in proximity to Valentia Island, Co. Kerry (5%), and across two areas on the north coast along the Co. Antrim coast (14%) (Figure 2a). Of the 1677 records, most (69%, *n* records = 1152) were collected more than 15 years ago (Figure 2b), which comprised the majority of records for hotspots on the northwest coast, including Clew Bay (96% of capture events for the grid cell), and on the north coast in proximity to Ballycastle (78%). With additional hotspots emerging only in recent years (≥2007) in areas along the south coast in proximity to Baltimore (+70 records in the corresponding 50 km^2^ polygon) and Union Hall (+241), and on the north coast in proximity to Larne, Co. Antrim (+84) (Figure 2c).

**TABLE 1:** The total number of unique taggers that contributed records per dataset per coast [the Inland Fisheries Ireland (IFI) mark‐recapture programme] and the number of records by coast [the Irish Specimen Fish Committee (ISFC) specimen dataset].

		Number of contributing IFI mark‐recapture taggers		Number of ISFC flapper skate records
Coast	North	All species	Flapper skate	
	East	93	2	4
	South	184	29	187
	Southwest	99	11	119
	West	83	14	68
	Northwest	82	10	17
	North	36	9	94

*Note*: The Irish coastline is defined by natural features, with the east coast spanning Carlingford Lough to Carnsore Point, the south from Carnsore Point to Mizen Head, the southwest from Mizen Head to Loop Head, the west from Loop Head to Erris Head, the northwest from Erris Head to Malin Head and the north from Malin Head to Carlingford Lough. Where an angler has submitted a single record to both IFI and ISFC, this record is counted as an IFI record.

**FIGURE 3:**
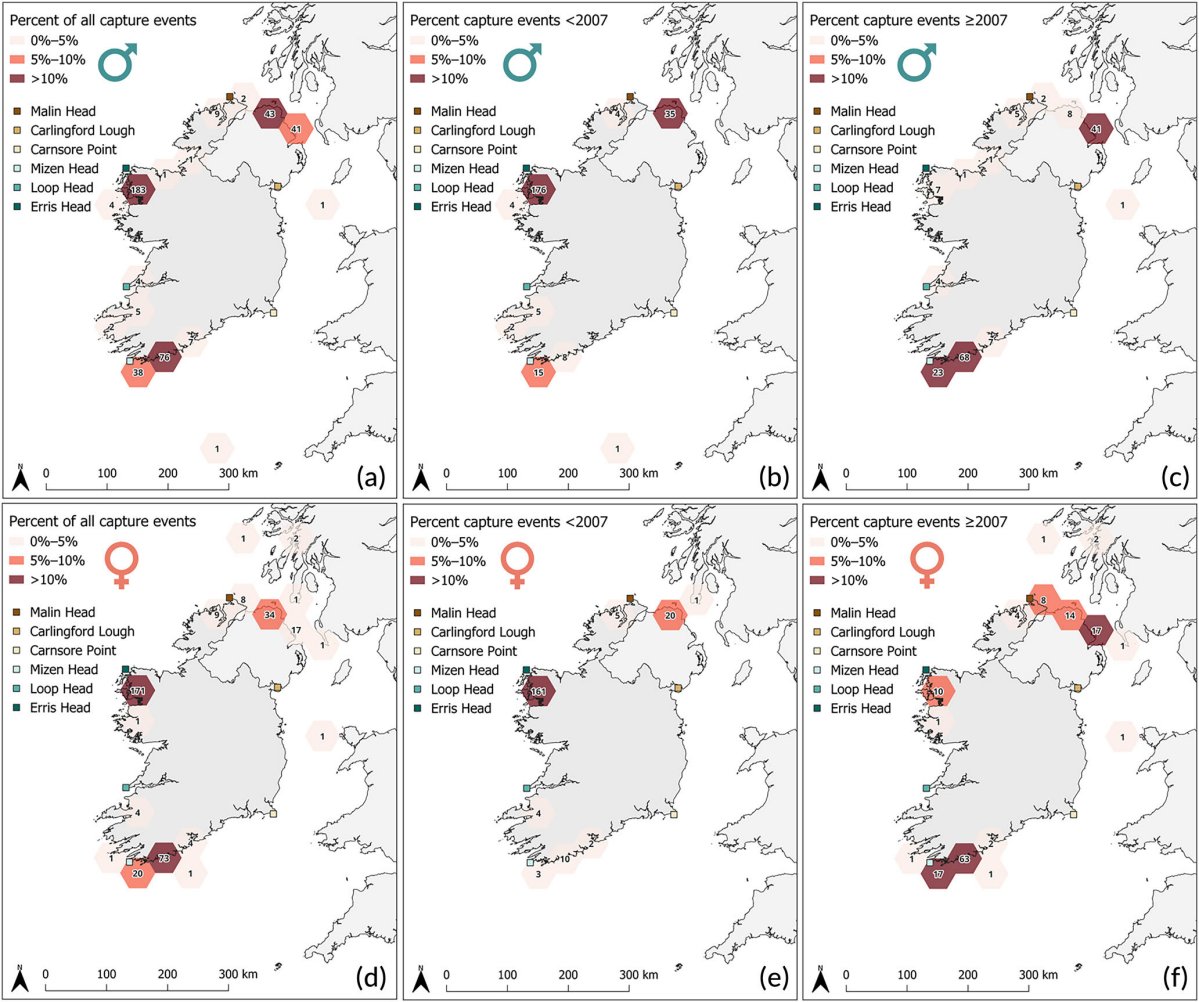
Spatial occurrence of mature flapper skate (*Dipturus intermedius*) recorded by the Inland Fisheries Ireland Marine Sportfish Tagging Programme and Irish Specimen Fish Committee specimen programme. A 50‐km^2^ hexagonal grid cell is coloured by the percentage of capture records (0%–5%, 5%–10%, >10%) relative to the number of records within a time frame. Cells are labelled as a count of capture events in a corresponding grid cell. (a) Male capture records from 1958 to 2006 (*n* = 250). (b) Male capture records from 2007 to 2022 (*n* = 168). (c) Female capture records from 1958 to 2006 (*n* = 206). (d) Female capture records from 2007 to 2022 (*n* = 143).

Of the 1677 capture events, 64% (*n* = 1073) had recorded fish sex and TL required to classify maturity. Of these 1073, 71% (*n* records = 767) of captured individuals were considered mature (*n* mature males = 418, *n* immature males = 14; *n* mature females = 349, *n* immature females = 306). A similar trend is seen in the spatial occurrence of mature flapper skate (Figure 2d), with hotspots identified across the northwest coast in proximity to Clew Bay, Co. Mayo (46% of all mature skate records), one area on the south coast in proximity to west Cork (20%) and on the north coast in proximity to Larne, Co. Antrim (10%). As seen in the full dataset (including fish below the maturity length threshold), the majority of mature skate records in hotspots across the west coast in Clew Bay, Co. Mayo (95% of all mature skate records for the polygon), and on the north coast in proximity to Larne, Co. Antrim (71%), are older than 15 years (Figure 2e). With additional hotspots of mature skate emerging only in recent years (≥2007) in two areas along the south coast in proximity to Union Hall and Baltimore (+131 and + 40 records in the corresponding 50 km^2^ polygons) and on the north coast in proximity to Ballycastle Port in Co. Antrim (+58) (Figure 2f). There were more records of mature males (54% of mature fish, *n* records = 418) than females. Similar distributions of males and females have been captured and reported over time, giving an approximate 1:1 sex ratio (Figure 3a–d).

### 3.2 Seasonality of capture

**FIGURE 4:**
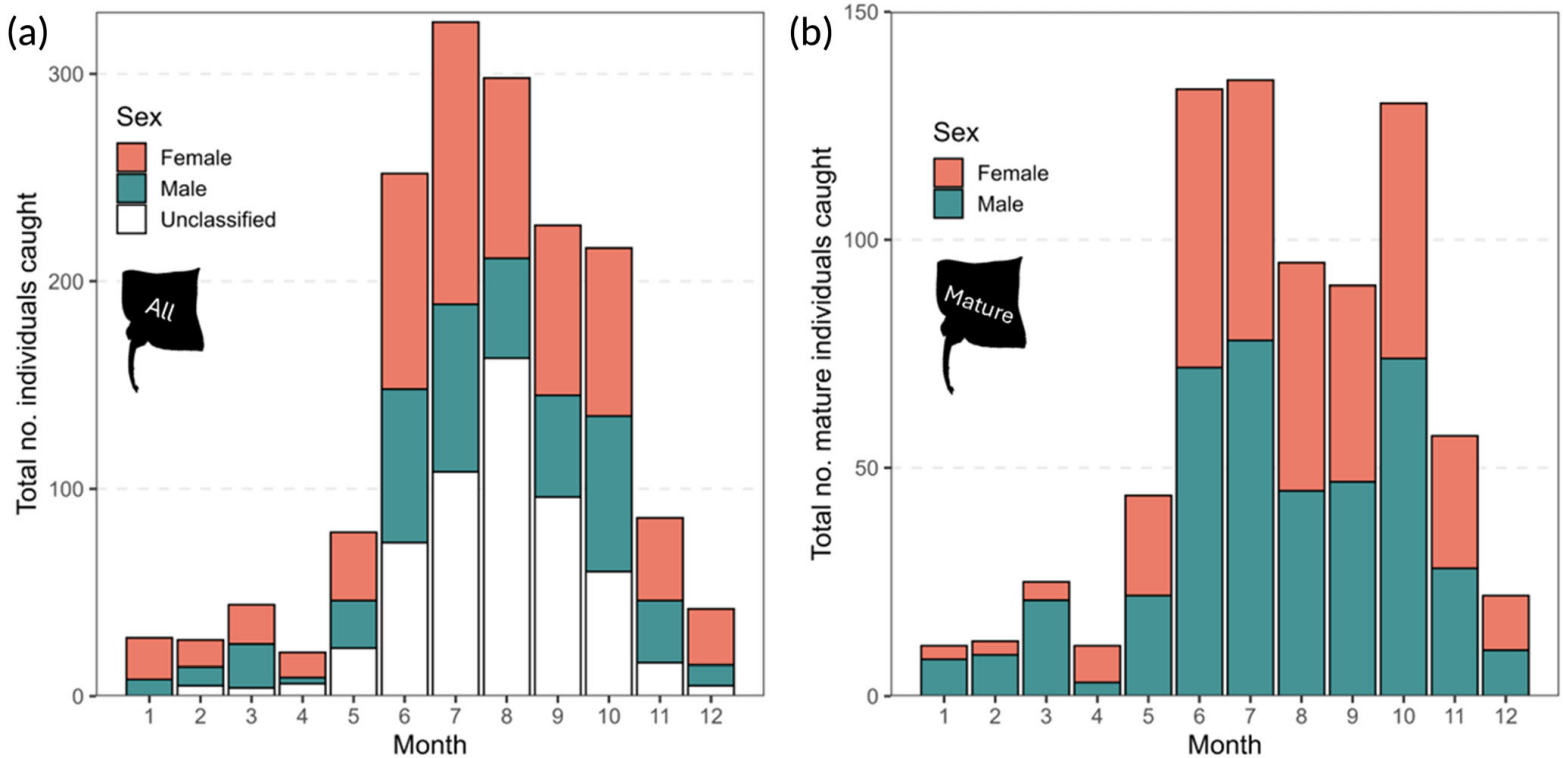
A stacked bar plot showing the seasonality of flapper skate (*Dipturus intermedius*) recorded by the Inland Fisheries Ireland Marine Sportfish Tagging Programme and Irish Specimen Fish Committee specimen programme. (a) All capture records from 1958 to 2022 with the month of capture recorded (*n* = 1645). These data include females (*n* = 654), males (*n* = 431) and unclassified individuals (*n* = 560). (b) Capture records of mature female flapper skates with a recorded month of capture and total length ≥ 203 cm (*n* = 348) and males with a total length ≥165 cm (*n* = 417).

Of the 1645 flapper skate records with an associated date of capture, most were caught between June and October (*n* records = 1318, 80% of all records) (Figure 4a), with the highest number of total capture records in July (20% of all catch data; *n* females = 136, *n* males = 81, *n* unclassified = 108). Mature skates (*n* records with an associated date of capture = 765) were most commonly caught in June (*n* records = 133, 17%), July (*n* = 135, 18%) and October (*n* = 130, 17%) (Figure 4b).

### 3.3 Dispersal

Of the 1061 unique skates recorded by the IFI mark‐recapture programme (1972–2022), there were 118 recapture events (10% recapture rate) of 103 unique fish [*n* females = 48 (47% of all recaptured skates), *n* males = 26 (25%), *n* unclassified = 29 (28%)]. With 89 individuals recaptured only once (*n* females = 44, *n* males = 21, *n* unclassified = 24), 13 were captured twice (*n* females = 4, *n* males = 5, *n* unclassified = 4), and 1 individual (sex unclassified) was tagged/recaptured three times within Clew Bay on the west coast. More mature females were recaptured once, than immature females [*n* mature female = 31 (65%), *n* immature female = 17 (35%)], with  slightly more on the second recapture [*n* mature female = 3 (60%], *n* immature female = 2 [40%]). Only mature male flapper skates were recaptured (*n* mature male skates in the entire IFI dataset = 418, *n* immature = 14). See Supplementary Material S1 for further information.

Time at liberty between capture events ranged from 0 to 11.5 years [mean days ± standard deviation (SD) = 531 ± 584], with 46% (*n* = 54) of recapture events less than 1 year since the last capture event, 30% (*n* capture events = 35) between 1 and 2 years and 25% (*n* = 30) of recapture events more than 2 years later. For female skates, time at liberty between capture events ranged from 0 to 11.5 years (mean days ± SD = 524 ± 680). Mature female skates (*n* recapture events = 33) spent on average 553 DAL (±812 SD), whereas immature female skates (*n* recapture events = 18) spent on average 529 DAL (± 375 SD). Furthermore, mature male skates (*n* recapture events = 31) spent on average 574 DAL (± 531 SD).

**FIGURE 5:**
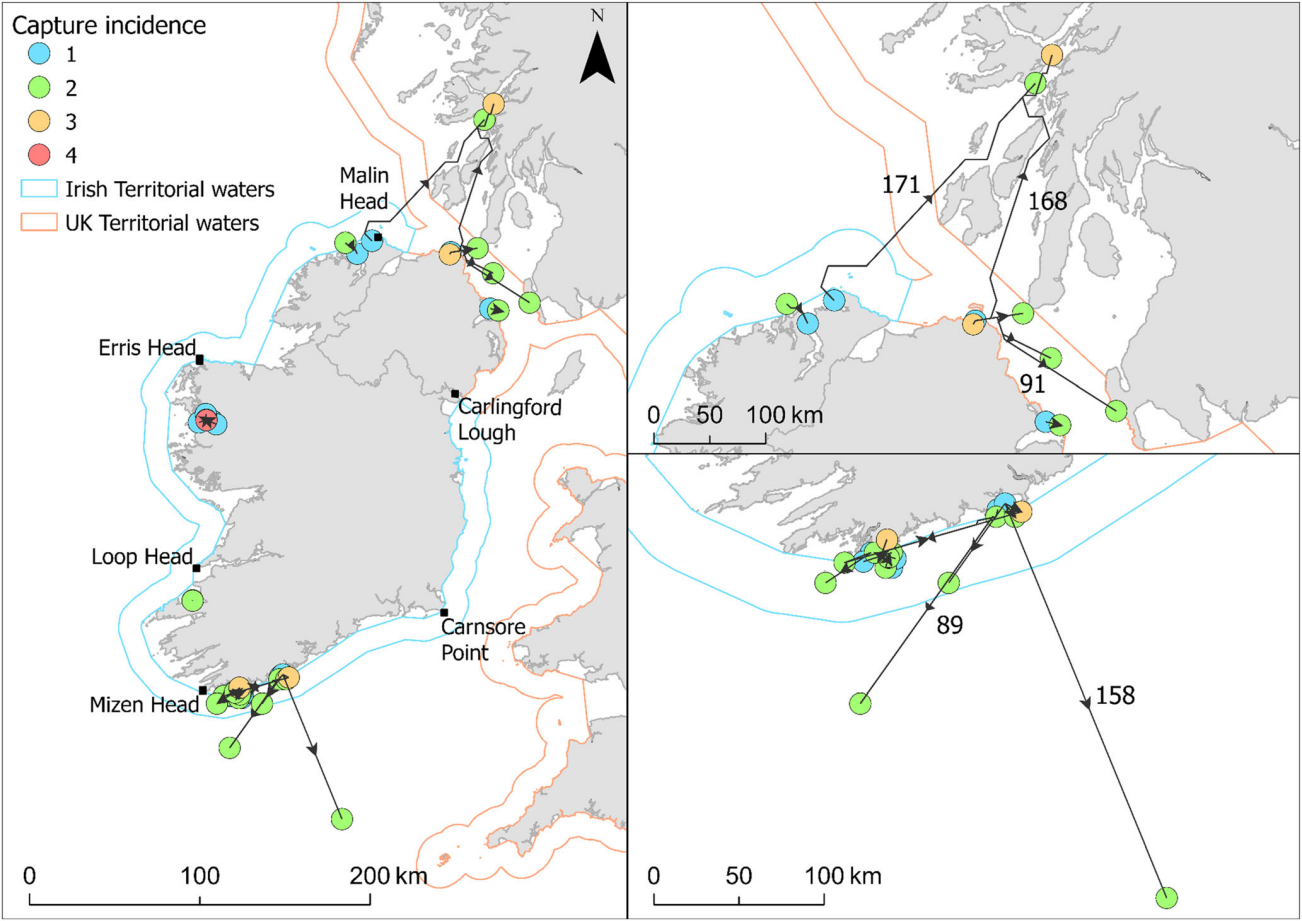
Capture and recapture locations of all flapper skate records from the Inland Fisheries Ireland mark‐recapture programme (*n* = 1188). Flapper skates were tagged across Ireland (left panel), with individuals in the north (top right panel) and the south (bottom right panel) displaying movements of over 100 km. Labels are visible on movement paths with a cumulative distance greater than 50 km (right panels).

Dispersal distances were typically less than 50 km from their original capture site (*n* individuals = 96, 93% of individuals; DAL range = 2–2473, mean DAL ± SD = 563 ± 534) (Figure 5). Of the 118 recapture events, 105 (89%) were within the same port where they were initially tagged. Mean dispersal distances were 12.9 km (±36.4 SD) for females, 12.3 km (±33.5 SD) for males and 7.6 km (±19.7 SD) for unclassified fish. Only three individuals (one immature female, one mature female and one mature male) had dispersal distances of over 100 km. The longest recorded dispersal distance was 171 km, observed in a mature female, and is one of two females tagged off the north coast (Malin Head, Co. Donegal; Ballycastle, Co. Antrim) that was subsequently recaptured within the Firth of Lorn (mature female DAL = 293; immature female DAL = 1421), specifically within the current boundaries of the Loch Sunart to the Sound of Jura MPA (Scottish Government, 2016).

## 4 DISCUSSION

Over 50 years of community science records highlight hotspots of flapper skates on the north, west and south coasts of Ireland. There are three main areas where mature skates were caught, including along the south coast in proximity to Union Hall, on the west coast in proximity to Clew Bay and on the north coast between Fair Head and Belfast Lough. Very few records were reported in the Irish Sea in concordance with their local extinction in the 1970s (Brander, 1981). Using a conservative length‐based filter, the present study offers insight into flapper skates that are likely over 10 years old (based on size‐age estimates provided in Régnier et al., 2021), including sexually mature flapper skates. Although mark‐recapture records are available for over 500 smaller individuals, given their grouping under the common skate complex, it was not possible to examine the movements of small juvenile flapper skate. Identification issues due to superficial physical similarities with other large *Dipturus* species across their range hamper the accuracy of current and historic species‐specific landings data available for stock management (Garbett et al., 2023; ICES, 2020, 2023). Given the size ranges of the four *Dipturus* species present in this area, it is improbable that any skate on or above 160 cm is anything but a flapper skate. Mark‐recapture data presented here suggest mature flapper skates and immature female flapper skates monitored for up to 11.5 years move within the boundaries of current ICES stock management units (Celtic Seas and western English Channel, Subarea 6 and divisions 7.a‐c and 7.e‐k; and the North Sea, Subarea 4), with connectivity observed between Northern Ireland and Scotland (five skates recaptured ≤10 km from the Scottish coast, 293–3522 DAL), as well as the south coast of Ireland and the Celtic Sea (three fish recaptured outside the Irish EEZ in the Celtic Sea, 301–981 DAL).

### 4.1 Spatiotemporal bias in catch records and the role of skipper effort

Catch records, as is commonplace in community science datasets, exhibit both spatial and temporal bias. Spatial bias occurs as taggers are more likely to engage in angling activities near their home port, in more accessible areas or at established and tested marks. For example, targeted skate angling along the east coast of Ireland is uncommon, possibly due to the perceived low likelihood of capture. This is exemplified by the number of taggers per coastline (Table 1), whereby there were only two IFI mark‐recapture taggers of the species on the east coast, conducted from trawlers. Whether effort or skate absence drives this trend is unclear. However, one of the most active commercial angling operators on the east coast uses large benthic‐set baits for shark angling, which would also likely attract skate. They are yet to report any *Dipturus* captures. Additionally, in this dataset, some taggers did not disclose their fishing marks and aggregated records to ports. In addition, where a skipper did not provide co‐ordinates but a descriptive location, capture co‐ordinates were drawn from a set list for a given area. Therefore, it was not appropriate to model dispersal distances in relation to potential drivers of movement. There is also a temporal bias in these datasets as fishing typically occurs during favourable weather conditions associated with early spring to autumn and targeting species when they are known to be more abundant (i.e., during summer). In this study, most flapper skates were caught between summer and early autumn, which coincides with the recreational elasmobranch fishing season in Irish waters (Casserly & Roche, 2023; Ryan et al., 2021).

Without data on skipper effort (i.e., no catch days and intentionality), it is difficult to ascertain whether the observed seasonality of flapper skate found here is due to presence/absence of skate or fluctuations in angler effort. However, angling for flapper skate uses a specific set‐up involving suitably rated angling gear, a large benthic‐set bait on a large hook from an anchored boat, and therefore, anglers typically capture this species (or other large elasmobranchs) intentionally. An example of both spatial and temporal bias is the role of skipper effort and involvement in the quantity and time span of catch records. The number of taggers involved in the IFI mark‐recapture programme has fluctuated over time, with some taggers contributing a disproportionate number of records (see Supplementary Material S2 for further information). For example, in Clew Bay, Co. Mayo, a single highly engaged volunteer tagged 393 skates between 1993 and 2007, which equates to 23% of the entire flapper skate dataset and 34% of older (≥15 y) records. Following a reduction in tagging effort in Clew Bay from 2007, the number of records for this area dropped from 575 (<2007) to 21 (2007–2022). Continued engagement and recruitment of active skippers and experienced anglers in community science programmes are critical to longitudinal monitoring and to establish whether a decline in records relates to localised population decline or programme engagement.

### 4.2 Distribution of occurrence records

Although angling effort for flapper skates is unevenly distributed across the Irish coastline (Table 1), community science records provide insight into the coastal occurrence of flapper skates in concordance with published work. Electronic tracking of flapper skate in the Sound of Jura, Scotland, found that depths of 20–75 m were used as frequently as depths of 100–175 m, notably by larger females (Thorburn et al., 2021). This contrasts with modelled occurrences of flapper skate using fisheries‐independent survey records, which suggests that depth and distance to coast are key determinants of flapper skate presence, with the highest presence at depths between 100 and 200 m (Régnier et al., 2024; Loca et al., 2025). Modelled fisheries‐independent trawl data suggest that presence of skate is highest 40–50 km from shore (Loca et al., 2025); however, modelled trawl datasets are often skewed towards areas away from the coastal fringe due to the reduced ability to sample in high rugosity habitat using mobile fishing gear deployed from large vessels. The presence of mature flapper skates closer to shore (as seen here), and their perceived use of coastal areas for egg‐laying (Dodd et al., 2022) and mating (Day, 1884), suggests that nearshore coastal areas are of vital importance and require further investigation. Given the multitude of pressures that occur within the nearshore environment (Marine Protected Area Advisory Group, 2024), focused research is needed into the function of perceived hotspots of skates along the south, west and north coasts of Ireland.

### 4.3 Seasonality of capture

Flapper skates were more commonly captured in summer to early autumn (June–October), and mature flapper skates were more commonly encountered in coastal waters in June, July and October. In the Loch Sunart to the Sound of Jura MPA, electronic tagging data suggest that depth use differs with sex, between size classes and with season (Thorburn et al., 2021). Electronically tagged skates were observed to occupy slightly shallower depths in winter and spring (220–248 m) compared to summer (265–312 m), which contrasts with the finding presented here that skates are more commonly encountered in coastal waters in summer and early autumn. Due to co‐ordinate uncertainty, it was not possible to examine capture depth due to the aggregation of records to ports. Given that fishing effort is lower in winter months in Ireland (Ryan et al., 2021), it is unclear whether this finding is related to increased skate presence or a result of heightened fishing efforts. However, the presence of females in coastal waters aligns with existing research that suggests that females exhibit high interannual seasonal site fidelity to shallow (<50 m) coastal waters during their potential egg‐laying period (Thorburn et al., 2021). Interestingly, two mature female flapper skates tagged on the north coast of Ireland were subsequently recaptured within the current boundaries of the Loch Sunart to the Sound of Jura MPA, an area specifically designated to conserve flapper skates through strict fisheries restrictions (Scottish Government, 2016). While these recaptures were before the MPA was established in 2014, this suggests that this area provides an essential refuge for part of the skate's range.

### 4.4 Recapture events and dispersal distances

Our results, consistent with mark‐recapture and electronic tagging studies, suggest that both immature and mature female skates, as well as mature male skates, exhibit site fidelity. Of the 1064 flapper skates conventionally tagged, 10% were recaptured. Although recapture rates of 10% are lower than published elsewhere, this may be due to the tagging of flapper skates in both transitional waters and open coastal environments (opposed to straits and sounds). In this study, all specimen claims and the majority of mark‐recapture records were submitted by recreational anglers within 12 NM of the coastline. Of the recaptured fish, almost all (94%) were recaptured less than 50 km from their original capture location, on average, 20 months after their first capture event. Whether short dispersal distances are due to bias towards coastal angling marks by recreational users (i.e., fishing closer to home ports) is unclear. However, multiple recapture events of skates two and three times in proximity to their original capture sites suggest that some individuals are resident to particular areas of the coast.

Similarly, mark‐recapture data collected in the Sound of Mull, Scotland, found that of 219 flapper skates conventionally tagged, 54 (28%) were recaptured and were caught at or near the release site (Little, 1995). In addition, Neat et al. (2015) found that of 280 tagged flapper skates tagged in the Sound of Mull between 2009 and 2021, 74 (26%) were recaptured, and 33 (12%) were recaptured on multiple occasions. Electronic tagging data have gleaned similar findings. Lavender et al. (2021) found that of 42 acoustically tagged flapper skates, almost half (48%, *n* = 16) exhibited residency periods of 3–12 months around acoustic receivers deployed in the Loch Sunart and Sound of Jura MPA (741 km^2^). Likewise, of four (*n* females = 3, *n* males = 1) flapper skates tagged in Loch Sunart, Scotland (assumed flapper skates based on reported total lengths of ≥160 cm), minimum dispersal distances ranged from 0.05–1.7 km (Wearmouth & Sims, 2009) with DAL ranging between 29 and 281 days. Additionally, all four flapper skates were reported recaptured within 2 km of their original tagging location. Existing research also suggests that flapper skates are capable of large‐scale movements. For example, Little (1995) found that two skates tagged in Scotland were reported 240 km (581 DAL) and 900 km (~1950 DAL) from their initial capture site. The latter individual was recaptured off the coast of Norway. These data, alongside electronic tagging data, suggest that populations comprise residents and transients (Lavender et al., 2021), with residency differing between demographic groups and sexes (Neat et al., 2015).

Females were more commonly recaptured than males (47% of recaptured fish vs. 25%). It should be noted that more females than males were present in the filtered mark‐recapture dataset [*n* females = 555 (59% of skates with sex classified), *n* tagged males = 379 (41%)]. Additionally, although a 1:1 ratio of sexually mature skate is observed in the entire (occurrence) dataset, and across hotspots of mature skate, including Clew Bay, Co. Mayo, and Union Hall, Co. Cork, anecdotal evidence suggests particular sexes are found more commonly in certain areas (at a higher resolution attainable than from this dataset). For example, off Courtmacsherry, Co. Cork, targeted angling efforts have produced a ~ 1:2 ratio of males to females (*n* total animals = 36, 2022–2024). A conservative filter was used to identify sexually mature skates based on the ultrasound and reproductive hormone work presented in Thorburn et al. (2023). This is based on reproductive hormone concentrations (oestradiol, testosterone and progesterone) of 84 flapper skates (*N* male = 47, *N* females = 36) captured and sampled in two locations in west Scotland between 2018 and 2021. Whether there are regional differences in age‐at‐maturity is not yet known. However, given that there is connectivity between populations on the north coast of Ireland and Scotland (Schwanck et al., 2023), the assumption of similar sizes at maturity between these areas is likely appropriate. Further work is needed to confirm the drivers of inshore space use of flapper skates, particularly given the diversity of activities occurring in shallow waters (Marine Protected Area Advisory Group, 2024), likely coinciding with critical habitats such as egg nurseries. Targeted research at remaining skate hotspots using tools such as electronic tagging alongside integrative tools (including, but not limited to, genetic sampling and reproductive hormone analysis) can identify potential drivers of residency and space use, dispersal and population mixing and replenishment (Lavender et al., 2021; Schwanck et al., 2023; Thorburn et al., 2023). Given potential sex‐based differences in the movement ecology of flapper skates, collecting accurate sex‐based classifications is key and should be prioritised in future community science data generation.

A conservative length‐based filter was used to identify flapper skate (≥160 cm) and mature male skates (≥165 cm). Therefore, most male skate records examined here were classified as mature (*n* mature male capture events = 418, equating to 97% of male records where maturity could be classified) and immature male skate movement could not be reliably examined. In line with the findings presented here, existing research suggests that mature males exhibit short periods of residency and multi‐annual site fidelity (Lavender et al., 2021).

### 4.5 Mark‐recapture data assumptions

Mark‐recapture relies on high retention of the selected tag type to ensure that the data generated are a result of recapture potential. Over time, the type of tag issued and used by volunteers has shifted from jumbo tags, Peterson discs and roto tags to dart tags owing to concerns over tag suitability, that is, biofouling, tag retention and tag‐related wounds (O'Reilly personal communication). Consequently, the application technique and position of the tag relative to the animal have also shifted. Several individuals tagged within the investigated dataset had markings consistent with previous tagging events, for example, a hole in one of its dorsal fins typical of a Jumbo tag insertion, suggesting the tag had been shed. This may have led to overestimating ‘unique’ individuals captured and underestimating how many individuals had been recaptured over time. Recent work has found that flapper skates have distinct dorsal spot patterns that can be used for photo identification (Benjamins, Dodd, et al., 2018; Benjamins, Fox, et al., 2018). Therefore, this issue could be ameliorated by encouraging taggers and claimants to submit photos alongside records and even substitute mark‐recapture tagging. A shift to photo‐based identification over traditional mark‐recapture also has the potential to enable data pooling from public sources, including social media. Pairing this method with minimally invasive genetic sampling techniques (i.e., using iDNA; Pascual et al., 2016) has the potential to bolster existing datasets and provide critical information on population demographics and mixing needed for devising effective conservation management strategies. A side‐by‐side comparison of these methods could be used to assess their potential.

## 5 CONCLUSION

Community science data, such as mark‐recapture and specimen fish records, offer an opportunity to engage with the fishing community, can provide a low‐cost data generation tool and serve as a platform to promote conservation and best‐practice approaches. The IFI mark‐recapture programme was originally designed for this purpose. Species‐specific identification is critical to generating effective fisheries and conservation management strategies; therefore, for morphologically similar species, additional evidence is needed (i.e., genetic material from the animal), particularly to bridge the gap in our understanding of all life stages. Further research is needed to investigate the movement of juvenile flapper skates to identify potential population bottlenecks in the populations of this Critically Endangered species. Additionally, area‐specific studies on flapper ecology and the drivers of hotspots around the coastline would help elucidate the presence of sensitive areas that could benefit from spatial protection, which have been proven successful for providing vital refugia for certain life stages of flapper skate (Régnier et al., 2024; Schwank et al., 2024).

## AUTHOR CONTRIBUTIONS

Danielle L. Orrell, William Roche and Thomas K. Doyle conceived the study. Ciara Wögerbauer and Shane O'Reilly collated and digitised the Inland Fisheries Ireland records. William Roche collated and digitised the Irish Specimen Fish Committee records. Danielle L. Orrell performed the data analyses. Danielle L. Orrell wrote the manuscript with contributions from Thomas K. Doyle, Ciara Wögerbauer, Shane O'Reilly and William Roche. All authors read and approved the final manuscript.

## FUNDING INFORMATION

D. L. Orrell is funded by the Sustainable Energy Authority of Ireland under the SEAI Research, Development & Demonstration Funding Programme 2021, grant number 21/RDD/670. This is publication #2 of the SEAI‐funded CETUS Project.

## Supporting information


**Data S1.** Supporting information.

## Data Availability

The data that support the findings of this study are available from two separate databases. The Inland Fisheries Ireland Marine Sportfish Tagging Programme dataset is available upon request from info@fisheriesireland.ie, with data available from https://urldefense.com/v3/__https://opendata‐ifigeo.hub.arcgis.com/search?categories=*252Fcategories*252Fmarine__;JSU!!N11eV2iwtfs!oF9LCs5uNYjNdS4IrSlLq0kcEFWrD7TQ3ChZ3VxB97V68BJB_FwVPl0AOEmo4TPitOdJyFgSQU5R(Notes)nbsp, and the Irish Specimen Fish Committee dataset is available upon reasonable request to isfc@fisheriesireland.ie.
