# Supplementary material for: Community science datasets identify the spatial occurrence and hotspots of flapper skate ( Dipturus intermedius )
Source: J Fish Biol. 2025 Oct 9;108(2):446–57. doi: 10.1111/jfb.70248 (PMC13052455; doi:10.1111/jfb.70248)
Supplement: Supplementary file 1 — Data S1. Supporting information. [file JFB-108-446-s001.docx]

**Community science datasets identify the spatial distribution and hotspots of flapper skate (*Dipturus intermedius*)**

**Supplementary material**

**Journal of Fish Biology**

Danielle L. Orrell*, Ciara Wögerbauer, Shane O’Reilly, Thomas K. Doyle, William Roche

***Author for correspondence**
Danielle L. Orrell - [dorrell@ucc.ie](mailto:dorrell@ucc.ie) School of Biological, Earth and Environmental Sciences, University College Cork, Distillery Fields, North Mall, Cork, Ireland. MaREI, The SFI Research Centre for Energy, Climate and Marine, Beaufort Building, Environmental Research Centre, University College Cork, Ringaskiddy, Cork, Ireland. ORCID ID: 0000-0002-9449-3920.

**S1. Recapture events**

Further information on recapture events recorded by the Inland Fisheries Ireland mark-recapture program is provided below. Note that in some cases, the skate total length was recorded in inches in the original paper logbook and later converted to centimetres during digitisation of records.

Table S1. One flapper skate was recaptured three times; this was a fish with an unclassified sex. Animal ID refers to the unique, indexed animal ID. Days at liberty (DAL) and total length (rounded to the nearest centimetre) are also provided.

| Animal ID | Capture event | Capture locale | Capture date | DAL (d) | TL (cm) |
| --- | --- | --- | --- | --- | --- |
| 003742 | 1 | Clew Bay, Co. Mayo | 1996-06-18 | - | 202 |
|  | 2 | Clew Bay, Co. Mayo | 1996-08-19 | 62 | 210 |
|  | 3 | Clew Bay, Co. Mayo | 1997-08-17 | 425 | 211 |
|  | 4 | Clew Bay, Co. Mayo | 1998-08-27 | 375 | 212 |

Table S2. Flapper skate recaptured twice (*n* = 12, excludes individual recaptured a third time). Animal ID refers to the unique indexed animal ID with fish sex given in brackets as either female (F), male (M) or unclassified (U). Days at liberty (DAL) and total length (rounded to the nearest centimetre) are also provided. In cases where the total length is only provided on capture or recapture, a single value may only be provided.

| Animal ID | Capture event | Capture locale | Capture date | DAL (d) | TL (cm) |
| --- | --- | --- | --- | --- | --- |
| 026390 (U) | 1 | Clew Bay, Co. Mayo | 2005-11-05 | - | 168 |
|  | 2 | Clew Bay, Co. Mayo | 2005-11-05 | 0 |  |
|  | 3 | Clew Bay, Co. Mayo | 2006-09-30 | 329 |  |
| 025489 (F) | 1 | Ballycastle, Co. Antrim | 2003-02-15 | - | 178 |
|  | 2 | Ballycastle, Co. Antrim | 2003-02-20 | 5 | 178 |
|  | 3 | Ballycastle, Co. Antrim | 2005-01-22 | 707 | 195 |
| 022441 (U) | 1 | Clew Bay, Co. Mayo | 2000-10-01 | - | 185 |
|  | 2 | Clew Bay, Co. Mayo | 2002-10-17 | 609 | 187 |
|  | 3 | Clew Bay, Co. Mayo | 2005-06-17 | 974 | 191 |
| 026763 (F) | 1 | Ballycastle, Co. Antrim | 2006-07-08 | - | 193 |
|  | 2 | Ballycastle, Co. Antrim | 2006-08-06 | 29 |  |
|  | 3 | Mull, Scotland | 2010-05-29 | 1421 |  |
| 036816 (M) | 1 | Clew Bay, Co. Mayo | 1994-09-05 | - | 188 |
|  | 2 | Clew Bay, Co. Mayo | 1994-10-01 | 26 | 188 |
|  | 3 | Clew Bay, Co. Mayo | 1998-09-08 | 1444 | 194 |
| 014036 (F) | 1 | Clew Bay, Co. Mayo | 1993-09-16 | - | 198 |
|  | 2 | Clew Bay, Co. Mayo | 1994-10-01 | 380 | 198 |
|  | 3 | Clew Bay, Co. Mayo | 1995-05-15 | 226 | 198 |

| Animal ID | Capture event | Capture locale (port) | Capture date | DAL (d) | TL (cm) |
| --- | --- | --- | --- | --- | --- |
| 022377 (M) | 1 | Clew Bay, Co. Mayo | 2002-05-29 | - | 193 |
|  | 2 | Clew Bay, Co. Mayo | 2003-08-15 | 443 | 196 |
|  | 3 | Clew Bay, Co. Mayo | 2005-06-02 | 1100 | 198 |
| 022376 (M) | 1 | Clew Bay, Co. Mayo | 2002-05-28 | - | 193 |
|  | 2 | Clew Bay, Co. Mayo | 2003-11-18 | 539 | 197 |
|  | 3 | Clew Bay, Co. Mayo | 2005-06-10 | 1109 | 199 |
| 023086 (F) | 1 | Clew Bay, Co. Mayo | 2002-06-18 | - | 198 |
|  | 2 | Clew Bay, Co. Mayo | 2000-06-22 | 4 | 198 |
|  | 3 | Clew Bay, Co. Mayo | 2005-06-18 | 1096 | 203 |
| 023813 (M) | 1 | Baltimore, Co. Cork | 2004-08-01 |  | 173 |
|  | 2 | Baltimore, Co. Cork | 2006-08-02 | 731 |  |
|  | 3 | Courtmacsherry, Co. Cork | 2008-07-15 | 1444 | 206 |
| 014713 (U) | 1 | Clew Bay, Co. Mayo | 1997-10-07 | - | 212 |
|  | 2 | Clew Bay, Co. Mayo | 1994-09-20 | 332 | 215 |
|  | 3 | Clew Bay, Co. Mayo | 1995-08-08 | 1113 | 215 |
| 018765 (F) | 1 | Clew Bay, Co. Mayo | 1998-11-14 | - | 203 |
|  | 2 | Clew Bay, Co. Mayo | 1999-08-25 | 284 | 221 |
|  | 3 | Clew Bay, Co. Mayo | 1999-07-29 | 257 | 230 |

Table S2 cont.

**S2. Skipper effort over time**

To investigate skipper effort over time, Inland Fisheries Ireland mark-recapture programme records were split into ‘old’ (1957-2006) versus ‘new’ (the last 15 years; 2007-2022). Data were visualised and inspected to ascertain the relative effort of skippers by area over time (Fig. S1). Of the 45 listed skippers involved between 1957 and 2006, the majority of data were collected in Clew Estuary, Co. Mayo by a single skipper (constituting 34% of old records, *n* records = 393). Very few records (*n* records = 25) for this area in recent years after the skipper reduced their tagging effort from 2007 onwards. Newer records were recorded by 29 skippers, with three individuals submitting 35% of all new records all in proximity to Co. Cork (Skipper “1” in Union Hall, Co. Cork: *n* records = 70; skipper “2” in Baltimore, Co. Cork: *n* records = 58; skipper “3” in Seven Heads, Co. Cork: *n* records = 56).


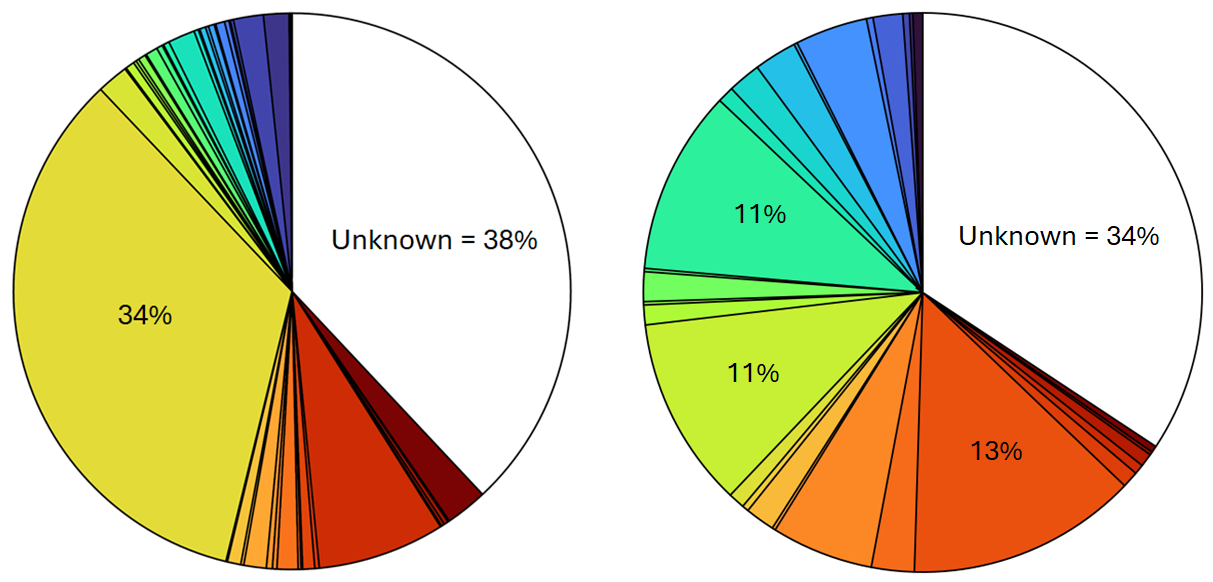
Figure S1. The proportion of flapper skate records contributed by skippers (*N* total skippers = 65) over time. Unknown skippers describe records where no skipper name is provided. Labels are displayed where a single skipper’s contribution exceeds 10% of the total records. A. All records between 1957 and 2006 (*n* records = 1152, *n* skippers = 45). B. All records between 2007 and 2022 (*n* records = 525, *n* skippers = 29).
